# A case of large-cell undifferentiated carcinoma of the bladder

**DOI:** 10.1093/jscr/rjad656

**Published:** 2023-12-06

**Authors:** Ali Zare, Omid Aminirad, Fariba Binesh, Elahe Jafaripoor, Farzad Moloudi, Behzad Narouie, Mohadese Ahmadzade

**Affiliations:** Department of Urology, Shahid Sadoughi University of Medical Sciences, Yazd, Iran; Department of Urology, Shahid Sadoughi University of Medical Sciences, Yazd, Iran; Department of Pathology, Shahid Sadoughi University of Medical Sciences, Yazd, Iran; Department of Pathology, Shahid Sadoughi University of Medical Sciences, Yazd, Iran; Department of Radiology, Urmia University of Medical Sciences, Urmia, Iran; Department of Urology, Zahedan University of Medical Sciences, Zahedan, Iran; Urology and Nephrology Research Center, Department of Urology, Shahid Beheshti University of Medical Sciences, Tehran, Iran; Urology and Nephrology Research Center, Department of Urology, Shahid Beheshti University of Medical Sciences, Tehran, Iran

**Keywords:** large-cell undifferentiated carcinoma, urinary bladder tumor, infiltrating urothelial carcinoma, neuroendocrine carcinoma

## Abstract

Large-cell undifferentiated carcinoma of the urinary bladder is an extremely rare and aggressive neoplasm. We present a unique case of painless gross hematuria and a past surgical history of cystolithotomy. The patient underwent transurethral resection of the bladder tumor, which revealed high-grade urothelial cell carcinoma with lamina propria involvement. Subsequent radical cystoprostatectomy with orthotopic neobladder urinary diversion and pelvic lymphadenectomy was performed, and the postoperative pathologic examination indicated large-cell undifferentiated. This case report highlights the importance of accurate diagnosis and management for this rare malignancy and adds to the limited existing literature on Large-cell undifferentiated carcinoma.

## Introduction

Large-cell undifferentiated carcinoma (LCUC) is a rare and aggressive histological subtype of malignant neoplasms that mostly originate from the salivary glands and lungs. Primary LCUC of the urinary tract is an extremely rare neoplasm, with only a few cases affecting the urinary bladder [1]. It is characterized by the presence of large, pleomorphic tumor cells with prominent nucleoli and abundant cytoplasm. Due to its rarity, the clinical presentation, diagnostic challenges, and optimal treatment strategies for LCUC remain poorly defined [2]. LCUC predominantly affects males between the ages of 61 and 87. In 2010 Beltran *et al*. reported eight cases of LCUC, with clinical presentations typically including hematuria, dysuria, or urinary frequency. These patients had a mean age of 74 years and exhibited a poor prognosis, with a mean survival of 11 months and a median survival of 7.5 months. Among the reported cases, six of the eight patients succumbed to the disease within 5–26 months [3]. Given the limited number of cases, there is a paucity of information on clinical behavior, diagnostic criteria, and optimal management strategies for LCUC.

## Case report

**Figure 1:**
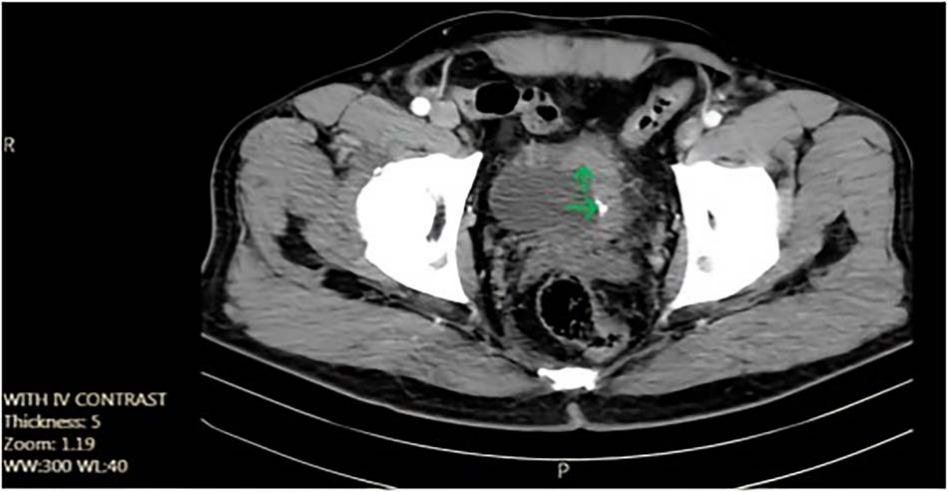
Axial pelvic CT scan shows large urinary bladder tumor (arrow).

We report the case of a 52-year-old non-smoker, non-addicted male with a past surgical history of cystolithotomy 30 years ago and no comorbidities. The patient presented to our center with painless gross hematuria without any obstructive or irritative urinary signs or symptoms. Initial ultrasonography revealed an 18 × 9 mm bladder stone, for which the patient was referred for cystolitholapaxy. Subsequent follow-up with ultrasonography and abdominopelvic computed tomography (CT) scan identified a 45 × 25 mm triangle laminated bladder stone (Fig. 1).

During cystoscopy, a large mass was observed in the bladder. Subsequently, transurethral resection of the bladder tumor was performed. The pathology report indicated high-grade urothelial cell carcinoma with lamina propria involvement. Chest and abdominopelvic CT scans and bone scans revealed no distant metastasis. The patient underwent neoadjuvant chemotherapy (four cycles of etoposide and cisplatin with etoposide 100 mg/m^2^ daily for 5 days and cisplatin 20 mg/m^2^ daily for 5 days administered every 3 weeks), which proved ineffective. Consequently, a radical cystoprostatectomy with orthotopic neobladder urinary diversion and pelvic lymphadenectomy was performed.

**Figure 2:**
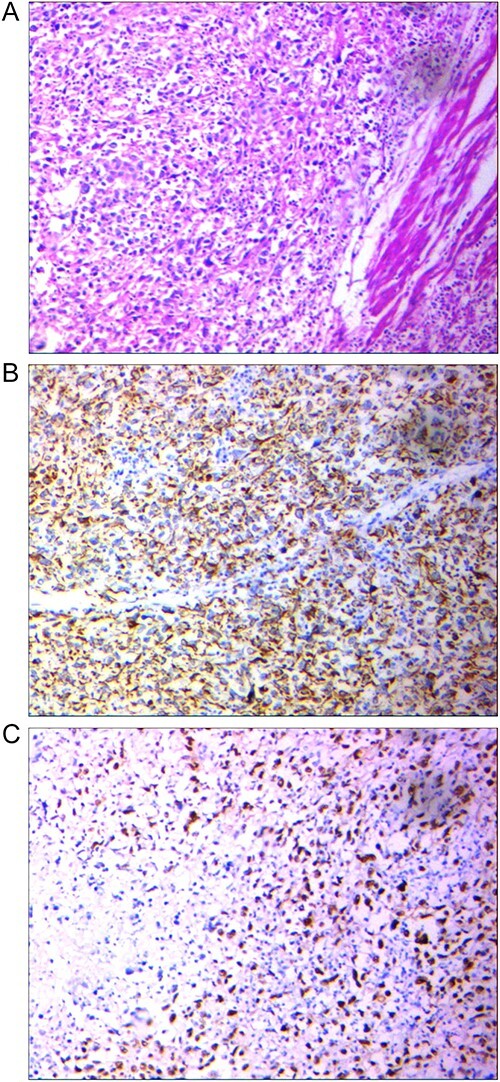
(A) Tumor with epithelial source, the proliferation of giant cell with polymorph nuclei and eosinophilic, Hematoxylin and eosin-stained section ×100. (B) Positive AE1/AE3 marker to point its epithelial source, IHC × 100. (C) Positive P53 point to High-grade tumor, IHC × 100.

The postoperative pathologic examination revealed high-grade urothelial carcinoma with the involvement of perivesical fat (pT3bN0M0, stage III). The tumor exhibited giant cell proliferation, polymorphic nuclei, and abundant eosinophilic to clear cytoplasm, along with invasion into the muscular bundle, necrosis foci, and calcifications (Fig. 2A), suggestive of large-cell undifferentiated carcinoma of the bladder. IHC examination demonstrated positivity for CK7, CKAE1/AE3 (Fig. 2B), and P53 (Fig. 2C), while P63, and synaptophysin were negative. At the 12-month follow-up, the patient remains disease-free.

## Discussion

Bladder cancer is the 4th most common cancer in men and the 6th overall [4], with urothelial carcinoma accounting for ~90% of all cases. Bladder cancer presents with a wide range of histological subtypes and clinical behaviors, posing challenges in diagnosis and management. Among these subtypes are the rare and aggressive neuroendocrine tumors, which originate from the neuroendocrine cells scattered throughout the urothelium.

Neuroendocrine tumors of the bladder are a diverse group of malignant neoplasms that originate from neuroendocrine cells and encompass several histological types. These histological types include small-cell neuroendocrine carcinoma, large-cell neuroendocrine carcinoma, carcinoid tumors, and paragangliomas. Each histological type of the tumor has distinct clinical and pathological features, which require different management strategies. For instance, small-cell neuroendocrine carcinoma has a poor prognosis and requires aggressive chemotherapy, while carcinoid tumors are less aggressive and may be managed with conservative surgical approaches. An accurate diagnosis of the histological type of the tumor is crucial for determining the appropriate treatment and improving the patients’ outcomes. LCUC of the urinary bladder, the focus of this case report, is an extremely rare form of neuroendocrine tumor that exhibits aggressive behavior [1].

LCUC was first described in 1997, and the current WHO classification defines it as a very rare form of infiltrating urothelial carcinoma. LCUC typically presents with hematuria, dysuria, or frequency and predominantly affects older men. In the study by Beltran *et al*., all eight patients, aged between 61 and 87 years, presented with hematuria. Half of these patients had no prior history of bladder cancer, while the others had a high-grade pathologic history [5]. In contrast, our case involved a younger patient, aged 52 years at the time of diagnosis, and his TUR pathology revealed high-grade urothelial carcinoma [6].

IHC analysis of LCUC commonly shows positivity for CK 8/18, AE1/AE3, CK7, CK20, uroplakin III, and GATA3 in 90% of cases, with some cases also exhibiting P63 positivity [3]. In our case, only AE1/AE3, CK7, and P53 were positive.

The prognosis of LCUC is generally poor, as the tumor is often identified at advanced stages and is associated with short survival, metastasis, or recurrence. In one study, 7 of 8 patients had lymph node metastasis, and 6 of 8 patients died from the disease within 5–26 months. Patients who underwent transurethral resection of bladder tumor and chemotherapy had a survival of 26 months [3]. In the present case, the patient has survived for 12 months at the time of writing this report.

The limited number of original reports on LCUC may be attributed to the confusing terminology and its rarity. The current case report, involving a patient diagnosed at a younger age of 52 years, adds to the body of knowledge on this rare malignancy, emphasizing the importance of accurate diagnosis and management, as well as the need for further research. Additional studies are necessary to better understand the clinical behavior, diagnostic criteria, and optimal treatment strategies for LCUC, ultimately aiming to improve patient outcomes and overall survival.

In conclusion LCUC is an aggressive tumor with frequent mitosis and a high proliferation index. Most patients have high-stage disease in presentation, and its prognosis is poor even with aggressive therapy.
